# Supplementary material for: miRNA Expression May Have Implications for Immunotherapy in PDGFRA Mutant GISTs
Source: Int J Mol Sci. 2022 Oct 14;23(20):12248. doi: 10.3390/ijms232012248 (PMC9603477; doi:10.3390/ijms232012248)
Supplement: Supplementary file 1 [file ijms-23-12248-s001.zip › ijms-1951583-supplementary.pdf]

Supplementary Material

1 Supplementary Figures and Tables

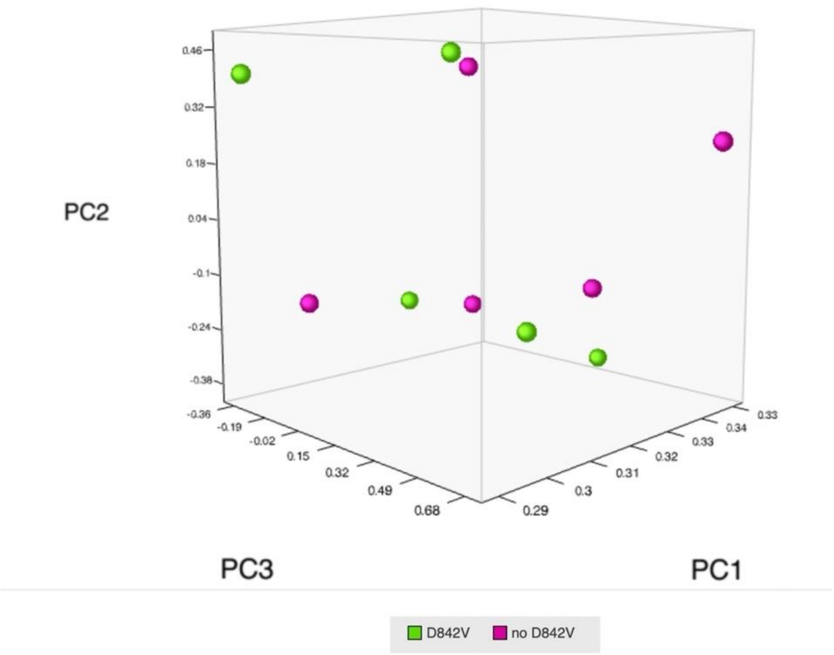

Figure S1. Global miRNA expression analysis. Principal component analysis of 10 samples analyzed via TLDA array. Each dot represents the collective expression of all microRNAs in one sample. Each color is indicative of a different GIST group.

**Supplemental Table S1: Reactome functional enrichment analysis**	
**Pathway**	**Adjusted P val**
**Top 30 GO: Biological Processes**	
Positive regulation of cellular metabolic process	8.76E-19
Positive regulation of metabolic process	3.61E-18
Regulation of cellular protein metabolic process	9.83E-15
Regulation of protein metabolic process	5.23E-14
Positive regulation of nucleobase-containing compound metabolic process	2.50E-12
Positive regulation of protein metabolic process	2.50E-12
Positive regulation of cellular protein metabolic process	9.26E-12
Regulation of apoptotic process	2.15E-11
Regulation of programmed cell death	2.76E-11

Interphase of mitotic cell cycle	2.76E-11
Cellular response to stress	2.76E-11
Interphase	3.39E-11
Response to abiotic stimulus	4.07E-11
Cellular protein metabolic process	4.21E-11
Regulation of molecular function	7.87E-11
Positive regulation of transcription from RNA polymerase II promoter	8.19E-11
Enzyme linked receptor protein signaling pathway	2.36E-10
Negative regulation of metabolic process	2.42E-10
Regulation of cellular metabolic process	2.46E-10
Epidermal growth factor receptor signaling pathway	2.81E-10
Positive regulation of transcription, DNA-dependent	3.14E-10
Interaction with host	3.14E-10
Positive regulation of RNA metabolic process	3.88E-10
Negative regulation of cellular metabolic process	3.88E-10
Regulation of catalytic activity	4.12E-10
Response to hypoxia	4.12E-10
Regulation of cell cycle	6.11E-10
Protein modification process	6.14E-10
Cell cycle	6.45E-10
Protein catabolic process	1.05E-09
**Top 30 GO: Molecular function**	
Enzyme binding	1.19E-16
Positive regulation of transcription, DNA-dependent	2.60E-09
Transcription factor binding	2.60E-09
Negative regulation of transcription, DNA-dependent	2.60E-07
Protein domain specific binding	3.04E-07
Transcription from RNA polymerase II promoter	6.57E-07
Kinase binding	6.77143E-06
Protein kinase binding	7.07778E-06
Protein binding, bridging	7.07778E-06
Binding, bridging	1.43636E-05
Protein N-terminus binding	1.43636E-05
Identical protein binding	4.01667E-05
Nucleotide binding	4.46923E-05
Chromatin binding	0.000139375
Protein complex binding	0.000139375
Protein C-terminus binding	0.000139375
Histone deacetylase binding	0.000205882
RNA binding	0.000744444

ATP binding	0.00165
Ubiquitin binding	0.00165
Ligase activity, forming carbon-nitrogen bonds	0.001804762
Adenyl ribonucleotide binding	0.001992308
Protein deacetylase activity	0.001992308
Adenyl nucleotide binding	0.001992308
Acid-amino acid ligase activity	0.001992308
Structure-specific DNA binding	0.001992308
Single-stranded RNA binding	0.002463333
Protein phosphatase binding	0.002463333
MAP kinase kinase kinase activity	0.002463333
Small conjugating protein binding	0.002463333
**Top 30 GO: Cellular component**	
Cytosol	1.73E-22
Macromolecular complex	6.87E-14
Nucleoplasm	6.87E-14
Nuclear part	3.93E-12
Nuclear lumen	5.84E-12
Protein complex	7.82E-12
Membrane-enclosed lumen	1.14E-11
Organelle lumen	1.54E-11
Nucleus	1.01E-10
Nucleoplasm part	0.00000622
Ubiquitin ligase complex	0.000101818
Spindle	0.000114167
Nuclear body	0.000326154
Histone deacetylase complex	0.000596429
Membrane-bounded vesicle	0.00230625
Eukaryotic translation initiation factor 3 complex	0.00230625
Ribonucleoprotein complex	0.003852941
Cytoplasm	0.003889474
Intracellular organelle part	0.003889474
Organelle part	0.0048
Transcription factor complex	0.005285714
Vesicle	0.005521739
Nucleolus	0.005521739
Spindle pole	0.006958333
Nuclear chromatin	0.00932
Spliceosomal complex	0.009538462
PML body	0.01525926
Non-membrane-bounded organelle	0.01874194

Intracellular non-membrane-bounded organelle	0.01874194
Spindle microtubule	0.01874194
